# The impact of intratumoral metabolic heterogeneity on postoperative recurrence and survival in resectable esophageal squamous cell carcinoma

**DOI:** 10.18632/oncotarget.14743

**Published:** 2017-01-19

**Authors:** Xinzhe Dong, Xiaorong Sun, Xianguang Zhao, Wanqi Zhu, Lu Sun, Yong Huang, Wenwu Li, Honglin Wan, Ligang Xing, Jinming Yu

**Affiliations:** ^1^ Department of Radiation Oncology, Shandong Cancer Hospital, Shandong University, Jinan, Shandong, China; ^2^ Key Laboratory of Radiation Oncology of Shandong Province, Shandong Academy of Medical Sciences, Jinan, Shandong, China; ^3^ Department of Radiology, Shandong Cancer Hospital and Institute, Jinan, Shandong, China; ^4^ Jinan University, Jinan, Shandong, China; ^5^ College of Physics and Electronic Science, Shandong Normal University, Jinan, Shandong, China

**Keywords:** positron emission tomography, FDG, esophageal carcinoma, intratumoral heterogeneity, prognosis

## Abstract

**Objective:**

To evaluate the impact of intratumoral metabolic heterogeneity measured by 18F-FDG PET imaging on postoperative recurrence and survival for patients with esophageal squamous cell carcinoma (ESCC).

**Results:**

AUC-CSH, metabolic tumor volume and pN-stage were significant prognostic factors for RFS. Additionally, tumor recurrence of the low AUC-CSH group (≤ 0.478) was 3 times higher than high group (*P* = 0.015). The median OS of patients with advanced AJCC stage or low AUC-CSH was also significantly shorter than that of patients with stage I & II or high AUC-CSH (*P* = 0.021, 0.009). Multivariate analysis identified the AUC-CSH to be the only significant risk factor for postoperative recurrence and overall survival in whole-group and stage III patients.

**Materials and Methods:**

116 ESCC patients who underwent staging 18F-FDG PET-CT scan and surgical resection were reviewed. The metabolic parameters were assessed as follows: maximum standardized uptake value (SUV_max_), metabolic tumor volume, and the area under the curve of the cumulative SUV-volume histogram (AUC-CSH), which is known to reflect the intratumoral metabolic heterogeneity. Regression analyses were used to identify clinicopathological and imaging variables associated with relapse-free survival (RFS) and overall survival (OS).

**Conclusions:**

Intratumoral metabolic heterogeneity characterized by AUC-CSH can predict postoperative recurrence and survival in patients with resectable ESCC.

## INTRODUCTION

Apart from the high incidence rate, esophageal carcinoma is one of the sixth leading causes of cancer-related deaths in the world [1]. It is known that Asian countries have the highest rates of esophageal cancer [2]. Compared with other types of esophageal carcinoma (mainly adenocarcinoma), esophageal squamous cell carcinoma (ESCC) has a poorer long-term survival. Esophagectomy has historically been considered the mainstay of treatment but has disappointing long-term outcomes [3].

Intratumoral heterogeneity is a well-recognized feature of malignancy tumor. A measurement that can quantify intratumoral heterogeneity may potentially predict outcome of ESCC . Positron emission tomography (PET) with 18F-fluorodeoxy glucose (18F-FDG) reflects important functional information on tumor cells. Before morphological changes occur, PET image shows metabolic abnormalities [4]. Currently, there is an increasing interest in using 18F-FDG PET images to quantify tumor tracer uptake heterogeneity and predicting treatment outcome non-invasively [5].

Recently, a innovative way to quantify intratumoral tracer uptake heterogeneity has been proposed by El Naqa et al. Cumulative SUV volume histogram (CSH), describes % of total tumor volume above % threshold of maximum SUV [6]. The area under the CSH (AUC-CSH) may be a quantitative parameter of metabolic heterogeneity. Our previous studies [7, 8] also showed that tumor FDG uptake heterogeneity accessed by PET image feature has the potential to detect advanced stage tumors in ESCC.

Kang. et al used AUC-CSH to define intratumoral metabolic heterogeneity of inoperable advanced non–small cell lung cancer (NSCLC), they found that AUC-CSH could predict disease progression after concurrent chemoradiotherapy (CCRT) [9]. Our previous study also found that the NSCLC patients with greater intra-treatment AUCH-CSH change had significantly longer survival time [10]. However, none of these studies have attempted to quantify 18F-FDG uptake heterogeneity using the AUC-CSH in patients with radical ESCC resection. Besides, the sample sizes of most similar studies were small, and the results of the prediction value of heterogeneity parameter remained undetermined.

The object of this research was to assess the possible usefulness of the intratumoral 18F-FDG uptake heterogeneity measured by PET imaging in prediction of postoperative recurrence and survival for patients with resectable ESCC.

## RESULTS

### Clinical characteristics

Table 1 shows patient demographics and Clinical Characteristics. The median age was 62.8 years (range 36.2–75.4), and they were followed for mean 39.6 months (range 4.6–68.0 months). Preoperative SUV_max_ ranged between 3.0 and 24.3, with median of 12.5. The median of MTV was 13.6 cm^3^ (range 3.2 cm^3^ −46.9 cm^3^). AUC-CSH ranged between 0.276 and 0.718, with median of 0.487. 27 patients received right-sided thoracotomy and upper midline laparotomy, other patients received left-sided thoracotomy for esophagectomy. All patients underwent R0 resection got a pathological stage.

**Table 1:** Patient demographics and clinical characteristics of 116 ESCC

Characteristics	No. of patients (%)	AUC-CSH (mean ± SD)	*P* value
Age			0.289
High (> 62.8)	52 (44.8%)	0.503 ± 0.108	
Low (≤ 62.8)	64 (55.2%)	0.472 ± 0.085	
Sex			0.894
Male	96 (82.7%)	0.467 ± 0.087	
Female	20 (17.3%)	0.585 ± 0.076	
Tumor location			0.524
Upper	16 (13.8%)	0.461 ± 0.082	
Middle	66 (56.9%)	0.591 ± 0.075	
Lower	34(29.3%)	0.474 ± 0.093	
ECOG performance status			0.068
0	13 (11.2%)	0.431 ± 0.062	
1	99 (85.3%)	0.495 ± 0.099	
2	4 (3.5%)	0.471 ± 0.096	
Preoperative comorbidity			0.195
No/minor comorbidity	104 (89.7%)	0.483 ± 0.098	
Major comorbidity	12 (10.3%)	0.521 ± 0.074	
SUVmax			0.068
High (> 12.5)	57(49.1%)	0.431 ± 0.060	
Low (≤ 12.5)	59(50.8%)	0.521 ± 0.062	
MTV			0.029
High (> 13.6 cm^3^)	53(45.7%)	0.407 ± 0.061	
Low (≤ 13.6 cm^3^)	63(54.3%)	0.593 ± 0.066	
Treatment modality			0.129
Surgery alone	9 (7.7%)	0.331 ± 0.042	
Surgery + adjuvant RT	38 (32.8%)	0.527 ± 0.124	
Surgery + adjuvant CT	14(12.1%)	0.532 ± 0.032	
Surgery+ adjuvant RT + CT	55 (47.4%)	0.463 ± 0.142	
pT stage			0.079
T1	22(14.3%)	0.521 ± 0.096	
T2	24 (20.2%)	0.522 ± 0.077	
T3	39 (43.3%)	0.458 ± 0.106	
T4	31 (22.2%)	0.493 ± 0.090	
pN stage			0.018
N0	39 (33.6%)	0.505 ± 0.082	
N1	29 (25.0%)	0.495 ± 0.114	
N2	31 (26.7%)	0.385 ± 0.096	
N3	17 (14.7%)	0.369 ± 0.065	
pM stage			0.092
M0	110 (94.8%)	0.490 ± 0.097	
M1	6 (5.2%)	0.422 ± 0.080	
pG stage			0.052
G1	24 (20.7%)	0.522 ± 0.077	
G2	62 (53.4%)	0.482 ± 0.091	
G3	30 (25.9%)	0.465 ± 0.104	
pAJCC stage			0.036
I	20 (17.2%)	0.538 ± 0.084	
II	22 (19.1%)	0.515 ± 0.094	
III	68 (58.6%)	0.469 ± 0.095	
IV	6 (0.5%)	0.382 ± 0.080	

The relationship between intratumoral heterogeneity status and clinicopathological characteristics is also summarized in Table 1. Lower AUC-CSH was significantly correlated with larger baseline MTV (*P* = 0.029), advanced postoperative AJCC stage (*P* = 0.018) and N stage (*P* = 0.036). On the other hand, there were no significant relationships between AUC-CSH value and age, gender, treatment modality, tumor differentiation, ECOG performance status, preoperative comorbidity, location, T stage or SUV_max_.

### Relapse-free survival and recurrence pattern

**Figure 1:**
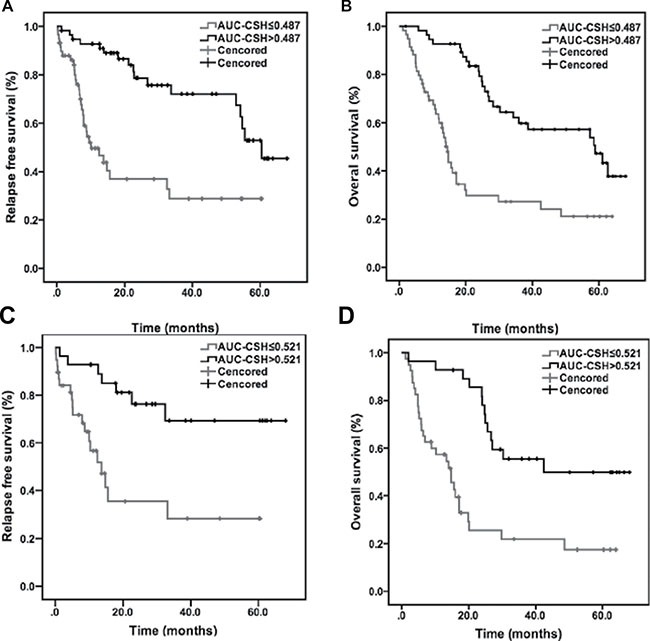
Kaplan–Meier plots for probability of RFS and OS Kaplan-Meier analyses of relapse free survival and overall survival according to AUC-CSH in whole group (116 patients) and subgroup (stage III, 58 patients).

With a 3-year RFS of 33.6%, the median RFS was 21.8 ± 14.1 months. Univariate survival analyses of RFS were shown in Table 2. Patients with lower value of AUC-CSH (≤ 0.487) relapsed quickly than higher AUC-CSH (> 0.487) (Median RFS: 13.4 months vs. 30.6 months, *P* = 0.002, Figure 1A). Larger MTV (> 13.6) was also associated with shorter relapse time (*P* = 0.033; Median RFS: 22.6 vs. 15.7 months). Besides, RFS was shorter in N2 and N3 patients (*P* = 0.027; Median RFS 18.8 vs. 25.6 months). As shown in multivariate COX regression analyses (Table 3), AUC-CSH was the only independent prognostic factors of RFS (*P* = 0.008; *HR*: 3.153 *95% CI*: 2.873–4.821).

**Table 2:** Univariate survival analyses of factors associated with RFS and OS

Variable	Relapse-free survival			Overall survival		
	HR	95%CI	*P* value	HR	95% CI	*P* value
Age (≥ 62)	2.987	0.839–8.583	0.542	3.453	0.452–6.217	0.361
Sex (male vs. female)	1.797	0.238–5.380	0.876	2.832	0.523–3.401	0.526
Histologic grade (G3 vs. G2, G1)	1.146	0.786–1.670	0.479	2.042	0.323–5.737	0.242
ECOG (2 vs. 1, 0)	2.165	0.829–5.438	0.326	1.883	0.783–3.264	0.682
Comorbidity (major vs. no, minor)	1.672	0.659–2.341	0.563	1.361	0.406–3.726	0.421
Tumor location (upper vs. other)	1.575	0.748–3.316	0.231	1.133	0.248–2.138	0.163
SUVmax (> 12.5)	3.505	0.233–12.761	0.365	4.221	0.683–7.294	0.365
MTV (> 13.6 cm^3^)	3.197	1.904–5.376	0.033	1.173	0.913–1.358	0.083
Surgery alone (vs. adjuvant therapy)	0.484	0.193–1.210	0.121	1.335	0.561–3.178	0.513
T4, T3 (vs. T2, T1)	0.237	0.032–1.771	0.161	2.433	1.003–5.899	0.049
N3, N2 (vs. N1, N0)	1.956	1.522–2.749	0.027	2.558	1.415–4.624	0.071
M1 (vs. M0)	1.134	0.587–2.193	0.708	1.567	0.808–2.033	0.101
AJCC stage III, IV (vs. I, II)	2.022	0.836–4.890	0.098	2.397	1.177–4.879	0.027
AUC-CSH (≤ 0.478)	3.529	2.118–6.892	0.002	2.745	1.506–4.938	0.004

**Table 3:** Multivariate survival analyses of factors associated with RFS and OS

Variable	Relapse-free survival		*P* value	Overall survival		*P* value
	*HR*	*95% CI*		*HR*	*95% CI*	
MTV (> 13.6 cm^3^)	1.236	0.719–1.852	0.433	1.573	0.629–1.953	0.326
N3, N2 (vs. N1, N0)	1.956	0.532–1.169	0.268	2.527	0.915–4.265	0.202
AJCC stage III, IV (vs. I, II)	2.193	0.431–4.245	0.118	1.236	0.749–3.126	0.241
AUC-CSH (≤ 0.478)	3.153	2.873–4.821	0.008	3.062	2.277–4.879	0.016

Recurrence was reported in 46 (39.7%) patients. On evaluating the recurrence pattern, we found 20 cases at locoregional level (12 relapse *in situ*, 5 mediastinal lymph nodes, and 3 abdominal lymph nodes), 17 distant cases, and 9 cases of mixed recurrences. Besides, tumor recurrence was observed in 35 (59.3%) of the low AUC-CSH group (≤ 0.478), which was 3 times higher than high AUC-CSH group (*P* = 0.015); In particular, locoregional recurrence showed a significantly higher rate in the low AUC-CSH group (*P* = 0.001). The frequencies of distant metastasis in the low AUC-CSH group were more than twice higher than in the high AUC-CSH group (Table 4).

**Table 4:** Patterns of recurrence

Recurrence pattern	No. of Patients (%)	High AUC-CSH (%)	Low AUC-CSH (%)	*P* value
Total recurrence	46 (39.7%)	11 (19.3%)	35 (59.3%)	0.015
Locoregional recurrence	20 (43.5%)	5 (8.8%)	15 (25.4%)	0.001
Distant metastasis	17 (36.9%)	5 (8.8%)	12 (20.3%)	0.023
Mixed recurrences	9 (19.6%)	4 (7.0%)	5 (8.5%)	0.279

### Overall survival analysis

In this study, 35.3 percent of patients were alive three years later, the median OS was 27.7 ± 20.2 months. Univariate analyses revealed that preoperative clinical features and treatment modality were not risk factors of OS (Table 2). On the other hand, lower AUC-CSH (≤ 0.487) was a significantly predictor of shorter OS (median OS: 18.4 vs. 37.2 months *P* = 0.004, Figure 1B). The overall survival of advanced AJCC stage (III-IV) patients was also shorter than stage I&II (median OS: 25.6 vs. 34.9 months *P* = 0.027). Besides, pT stage, pN stage and histologic grade were also not significant predictors of OS (Table 2). Median OS of patients with lower MTV (≤ 13.6) was not significantly longer than high MTV (> 13.6) (28.1 vs. 29.3 months *P* = 0.083).

Each of clinical and PET features entered multivariate analysis separately, for high degree of collinearity with each other. At multivariate Cox analysis for OS outcome (Table 3), it was found that the only independent predictive factor associated with decreased overall survival was low AUC-CSH (*P* = 0.016; *HR* = 3.062, *95% CI*: 2.277–4.879).

In the subgroup analysis of pathologic stage III patients, the median of AUC-CSH is 0.521. Compared with high AUC-CSH, patients with lower AUC-CSH value progressed quickly and had a significantly shorter survival time (*P* = 0.023 and *P* = 0.012, Figure 1C, 1D). After multivariable analysis, only AUC-CSH retained its significant predictive value for RPS (*P* = 0.032, *HR* = 2.586, *95% CI*: 1.952–4.273) and OS (*P* = 0.026; *HR* = 3.215, *95% CI*: 2.526–5.628). High pN-stage, MTV or AJCC stage (III_c_, III_b_ vs. III_a_) (*P* = 0.079, *P* = 0.143 and *P* = 0.239) were not independent prognostic factors for shorter RFS or poor OS.

## DISCUSSION

Personalized medicine is a goal in modern cancer therapy that aims for optimal treatment for an individual patient that is dependent on tumor characteristics in that individual [11]. Despite medical advances in recent decades for the treatment of esophageal carcinoma, long term survival rates are still low [12]. It is therefore important to identify better prognostic factors. This study showed that intratumoral metabolic heterogeneity assessed by AUC-CSH is a strong independent prognostic feature in patients with ESCC resection.

Our study analyzed clinical and pathologic features of 116 resectable ESCC, the prognostic value of AUC-CSH was confirmed. A lower baseline AUC-CSH value (higher metabolic heterogeneity status) was correlated with poor outcome. Besides that, in the subgroup (III stage) analysis, the AUC-CSH was also an accurate predictor of RFS or OS. As it is found that most esophagus carcinoma is not discovered until the advanced stages, the observation is more valuable. In addition to pathological AJCC stage, AUC-CSH might be a noninvasive quantitative biomarker to stratify patients with a worse outcome before treatment is initiated.

According to results of our previous [10] and current studies, AUC-CSH was related to AJCC stage. Moreover, multifactor analysis revealed that AUC-CSH was an independent prognostic factor of survival. This observation may have a reasonable explanation that reflects the inherent biologic characteristics of the tumors [13]. The patients with higher heterogeneous tumors fare more poorly than those with homogeneous tumors, and accompany with higher lymph node metastasis rate.

This result is not unexpected because intratumor heterogeneity may foster tumor adaptation and therapeutic failure through Darwinian selection [14]. Aggressive tumors grow faster and involve more intratumoral hypoxia [15], which leads to FDG accumulate differently in necrosis, inflammatory infiltration [16] and cancer stem cells [17]. As a result, intratumoral heterogeneity could be depicted in PET images through chaotic FDG uptake. As shown in our results, lower heterogeneity parameter correlated with larger MTV and advanced N stage. Similarly, one previous research of Kidd et al. also found that cervical pretreatment FDG-PET metabolic heterogeneity predict risk of lymph node involvement and pelvic recurrence [18]. Additionally, tumor recurrence observed in low AUC-CSH group was 3 times higher than high AUC-CSH group in our research.

The pretreatment larger MTV was associated with worse outcomes in certain cancers such as the lung [19], oral cavity carcinoma [20], and uterine cervical cancer [21] independent of disease stage. In this study, we also found smaller MTV (≤ 13.6) was associated with improved RFS in univariate analysis. However, AUC-CSH was the only independent predictor of RFS. Based on previous researches [7, 22], we know that tumor size was correlated with tracer uptake heterogeneity.

In another aspect, dynamic monitoring of MTV during treatment may be more valuable and practical in the clinic. Wei et al. found that a decrease of MTV during the early stage of CCRT was correlated with higher OS [23]. Tamandl et al. also concluded that volumetric changes induced by neoadjuvant chemotherapy are independent prognostic factors for survival in patients with radical esophageal cancer resection [24]. For R0 resection, MTV change can't be monitored in this research. However, we are conducting another prospective study to monitor more metabolic parameters’ change for unresectable ESCC.

Unlike in previous reports [25–27] on the correlation of SUV_max_ with treatment response and prognoses of various malignancies, we did not find any significance between SUV_max_ for the primary tumor and outcome in our cohort. Median value of SUV_max_ was used as the optimal cut-off for analyses in this study. Nevertheless, the cut-off value of SUV_max_ (14.9) was also estimated from ROC analysis in predicting tumor recurrence. Specificity (70%, 95% CI: 38.2%–89.6%) and sensitivity (52%, 95% CI: 35.1%–70.2%) were derived from AUC-ROC (0.572, *P* = 0.073), it also failed to show statistical significance in analyses. Possible explanations for the discrepancy between the results of previous studies and the present study would be heterogeneity of tumor character and election of treatment. Therefore, the result of the present study on the predictive role of SUV must be interpreted with caution. Another explanation could be that because ESCC originate from the mucosa, the status of the mucosa (inflammation or postbiopsy) might increase maximal SUV. Interestingly, all these findings could make us speculate that AUC-CSH might be not a simple surrogate of SUV and could be an independent factor.

Although heterogeneity can be associated with malignant behavior of the tumor, there's no universal definition. Including visual evaluation [28], AUC-CSH [6], and texture analysis [4], a few main parameters have been proposed to quantify metabolic heterogeneity. Visual assessment may be considered as a simple way of scoring intratumoral tracer distribution. However, it can be difficult to implement in clinics because of higher inter-observer variability. The accuracy and precision of texture analysis in clinical evaluation depends significantly on individual scanning protocols. Factors such as image acquisition, reconstruction and inherent image quality parameters may be important [29]. In comparison, the AUC-CSH index yields an intuitive and fairly robust tool for extracting information on the spatial gradient of the tumor heterogeneity as demonstrated by our results. Moreover, the sign of AUC-CSH index provides additional information on relative changes in SUV between the SUV-based tumor center and periphery.

The major disadvantages of current research are the retrospective design, the heterogeneity of the patients and treatments, and only primary tumor were analyzed. Besides, the process of ROI extraction was not full automation. Accurate segmentation technology could ensure the smooth implementation of multi-center studies. Ongoing studies will also explore the potential relationships between immunohistochemical staining patterns and intratumoural heterogeneity on functional imaging.

Despite the disadvantages, however, it was demonstrated that the preoperative intratumoral metabolic heterogeneity status characterized by AUC-CSH was reliably to identify the high-risk population for postoperative recurrence and short survival in patients with radical ESCC resection. Large-scale investigations should be conducted to determine the bias and variance in multicenter. In conclusion, AUC-CSH could be an important factor to be considered in the treatment planning and follow-up of patients with resectable ESCC.

## MATERIALS AND METHODS

### Patients

This study was approved by the institutional review board at Shandong Cancer Hospital. Informed consent was waived due to the retrospective design of the study. From the cancer registry at Shandong Cancer Hospital, we retrospectively analyzed one hundred and sixteen consecutive patients with previously untreated, biopsy-proven ESCC from March 2010 to March 2013. All the patients underwent esophagogastroduodenoscopy, endoscopic ultrasound, computed tomography scan of chest and upper abdomen, and 18F-FDG PET/CT scan before curative esophagectomy. For patients who have not received preoperative therapy, our institute has included postoperative fluoropyrimidine-based chemoradiation for patients with T3–T4 tumors, node-positive T1–T2 tumors, and selected patients with T2, N0 tumors with high-risk features. Locoregional recurrence was defined as occurring on an anastomosis site, the mediastinum, or the abdomen where lymph nodes were dissected. Distant recurrence was defined as those occurring outside the operative field, such as in the lung, brain, liver, adrenal glands, bone, or other location. Recurrence was diagnosed based on the PET/CT and chest CT results, and tissue biopsies were taken of suspected recurrent lesions if possible. Adjuvant therapy including radiotherapy (RT) and chemotherapy (CT) after initial curative esophagectomy was determined after discussion among the surgical, medical, and radiation oncologists. Clinical follow-up was done every 2–4 months during the first year, every 4–6 months during the next 2 years, and every year thereafter.

### 18F-FDG PET/CT Scan

All the 116 patients underwent the pre-treatment whole-body 18F-FDG PET/CT scan 1 week before the surgery. Patients were fasted for at least 8h prior to 18F-FDG PET/CT scanning, and the blood glucose level was < 1.4g/L before scans for all patients. The FDG PET/CT images were obtained using a GE Discovery LS system 60 minutes (range 55–70 min) after injection of 18F-FDG (4.4 MBq/kg) with a rigid protocol [30]. CT data were acquired first (120 kV and 90mA, no contrast enhancement). PET images were subsequently reconstructed with the built-in GE advance software, using the ordered subset expectation maximization (OSEM) algorithm with 2 iterations and 28 subsets, and a 5.0 mm full-width at half-maximum (FWHM) Gaussian post-filtering. The PET (128 × 128, pixels of 3.91 × 3.91mm, 4.25-mm slice thickness) and the CT (512 × 512, pixels of 0.98 × 0.98mm, 5.0mm slice thickness) images were systematically co-registered using the GE software.

### PET imaging analysis

Our previous study demonstrated that the tumor length at FDG PET image with the cutoff value of 2.5 was closest to the gross tumor length [31]. Based on this result, the regions equal or greater than SUV 2.5 were selected to automatically delineate region of interest (ROI). Two clinical oncologists with the help of a specialist radiologist adjusted manually by visual inspection of the primary tumor borders to avoid overlapping on adjacent FDG-avid structures or lesions. All metabolic parameters were subsequently extracted from this delineated volume. The SUVmax in each ROI was determined using the whole-body attenuation corrected image. The MTV was automatically generated from the ROI in cubic centimeters (cm3) using the Xeleris workstation.

**Figure 2:**
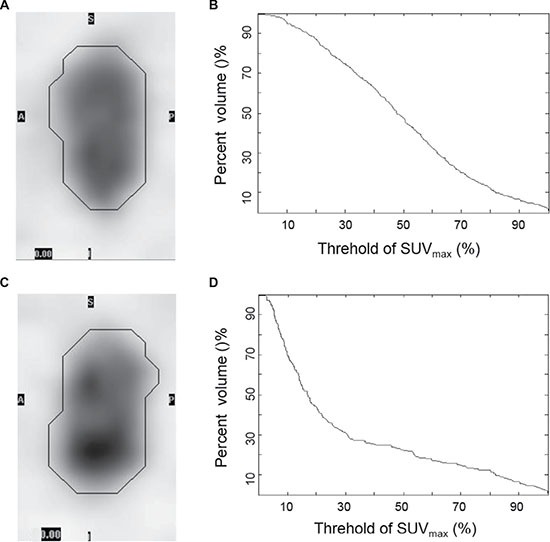
Typical examples of FDG uptake heterogeneity Tumor with a lower degree of heterogeneity (**A**) showed higher AUC-CSH (**B**) and tumor with a higher degree of heterogeneity (**C**) showed lower AUC-CSH (**D**).

Intratumoral metabolic heterogeneity was evaluated by the AUC-CSH, which was known to reflect the tumor heterogeneity [7, 13, 14]. These histograms are similar to dose-volume histograms frequently used in radiotherapy. CSH is normally obtained by plotting the percent volume of a tumor with an SUV above a certain threshold against that threshold, which is varied from 0 to 100% of SUVmax. The AUC of this plot (AUC-CSH) is a quantitative index of uptake heterogeneity, where lower values correspond with increased heterogeneity. Figure 2 shows two typical examples of FDG uptake heterogeneity, the metabolic tumor volume (black) was the ROI to be segmented and analyzed. All image processing process such as ROI segmentation, denoising and CSH extraction was performed using a code developed and implemented in-house at MATLAB (Mathworks Inc, Natick, USA).

### Study design and statistical analysis

To evaluate the prognostic value of intratumoral metabolic heterogeneity feature, Relapse-free survival (RFS) and OS was chosen as endpoints. RFS was calculated from the date of the operation to the date of the first recurrence or the last follow-up. OS was measured from the date of the operation to the date of death or the last follow-up. The statistics analysis was performed using SPSS for Mac (version 22, IBM). According to clinical characteristics, the AUC-CSH of various groups was expressed as the mean ± SD. The differences of AUC-CSH among subgroup were tested using one-way analysis of variance (ANOVA) or Kruskal–Wallis test. The SUV_max_, MTV, AUC-CSH and ages were analyzed as binary variables using the median values as cutoff levels, which is more than median as the high group and less than or equal to median as the low group. Tumor (T) classification, lymph node (N) classification, differentiated degree (G) and AJCC stage were analyzed as categorical variables. Survival probabilities were estimated using the Kaplan–Meier method, and the difference between survival curves in relation to low and high levels of each prognostic factors were tested using the log-rank test in the univariate analysis. Multivariate analysis was performed to identify the prognostic factors influencing RFS and OS using Cox proportional hazards regression model. Only significant variables after univariate survival analysis were included in multivariate survival analysis. All statistical tests were conducted at a two-sided level of significance of 0.05.
